# Partial Papillary Muscle Rupture after Myocardial Infarction and Early Severe Obstructive Bioprosthetic Valve Thrombosis: an Unusual Combination

**DOI:** 10.5935/abc.20180179

**Published:** 2018-09

**Authors:** Inês Silveira, Marta Oliveira, Catarina Gomes, Sofia Cabral, André Luz, Severo Torres

**Affiliations:** Centro Hospitalar do Porto, Porto - Portugal

**Keywords:** Myocardial Infarction/complications, Thrombolytic Therapy, Atrioventricular Block/complications, Pacemaker Artificial, Heart Arrest, Heart Rupture, Post-Infarction, Bioprosthesis

## Introduction

Mechanical complications after myocardial infarction (MI) have become uncommon since the introduction of primary angioplasty.^1^ They can lead to a rapid clinical deterioration and a fatal outcome, with patient's survival being depen­dent on their prompt recognition and intervention. We describe a case of two rare mechanical complications: a partial papillary muscle rupture after MI, followed by an early severe obstructive thrombosis of the implanted bioprosthetic valve.

### Case report

**Figure 1:**
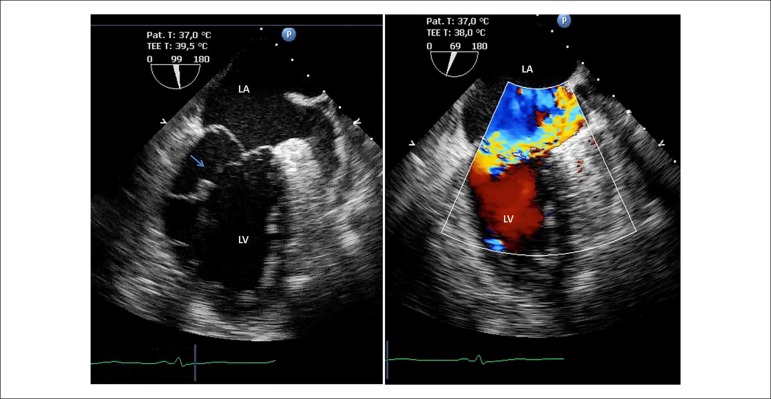
Transoesophageal echocardiogram two-chamber view showing a 9 mm disruption of the posteromedial papillary muscle (pointed with an arrow) consistent with a contained, but morphologically imminent rupture, leading to a broad posterior leaflet prolapse and a severe mitral regurgitation with an eccentric jet. LA: Left Atria; LV: Left Ventricle.

**Figure 2:**
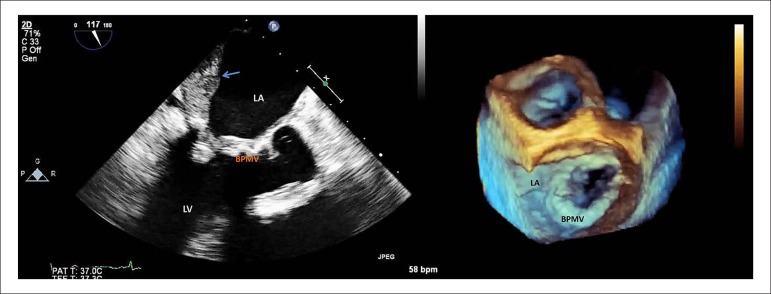
Transoesophageal echocardiogram long axis view and 3D zoom on face view of the mitral valve presenting a significant thickening of mitral bioprosthetic cusps due to thrombotic material deposition, leading to severe obstruction of the prosthetic valve and a large mural thrombus covering the left atrial posteroseptal wall (pointed with an arrow). BPMV: Bioprosthetic Mitral Valve; LA: Left Atria; LV: Left Ventricle.

We report a case of a 70 year-old male, with a history of dyslipidaemia and smoking habits, who suffered an inferior ST elevation myocardial infarction (STEMI). Given the impossibility to achieve a timely percutaneous coronary artery intervention, thrombolysis was performed within 4 hours of symptoms onset. Advanced atrioventricular block requiring a transcutaneous pacemaker occurred soon after, followed by cardiorespiratory arrest in ventricular fibrillation, which was reversed after one cycle of advanced life support. The patient was transported by airplane to a percutaneous coronary intervention (PCI)-capable centre. Coronary angiography showed a 50-60% stenosis in the proximal segment of the right coronary artery, which was treated with a bare metal stent. Echocardiography showed a moderate left ventricular systolic dysfunction (estimated ejection fraction of 35%), with inferior, inferolateral and inferoseptal akinesia and moderate mitral regurgitation. On the fifth day, the patient was transferred to our centre, after a 10-hour flight. On admission to intensive care unit, the patient was in cardiogenic shock with inotropes and non-invasive ventilation. A bedside transthoracic echocardiography revealed a severe mitral valve regurgitation of uncertain mechanism, along with moderate left ventricle systolic dysfunction and right ventricle systolic compromise. Additional characterisation by transoesophageal echocardiography revealed a 9 mm disruption of the posteromedial papillary muscle consistent with a contained, albeit morphologically imminent, rupture. The instability of the sub-valvular apparatus, leading to a broad posterior leaflet prolapse, caused severe mitral regurgitation with an eccentric jet with Coanda effect, reaching the left atria roof (Figure 1). The patient underwent urgent mitral valve replacement with a biological prosthetic valve (St. Jude #29), with preservation of anterior and posterior leaflets. The patient experienced a favourable post-operative recovery and was discharged 12 days after surgery with anticoagulant therapy for three months, in addition to dual antiplatelet therapy. On the fourth month after surgery, the patient initiated progressive heart failure symptoms (NYHA class III) without any further complaints. Additional transthoracic and transoesophageal evaluations were performed, revealing a significant restriction of the prosthetic mitral valve leaflets mobility due to thrombotic material deposition, leading to severe obstruction, with a mean gradient of 19 mmHg and an effective orifice area estimated by PISA method of 0.4 cm.^2^ Additionally, in continuity with the prosthesis, a large mural thrombus was present covering the left atrial posteroseptal wall (Figure 2). Urgent surgery, within twenty-four hours after diagnosis, was performed involving mitral bioprosthesis replacement with another biologic prosthesis with significant improvement in clinical status. After an extensive study, no evidence was found of atrial fibrillation or thrombotic disorders. Pathology examination of the excised prosthetic material confirmed prosthetic thrombosis, with no signs of endocarditis.

## Discussion

In the current era of early mechanical reperfusion, the incidence of papillary muscle rupture (PMR) after MI has decreased, being less than 0.5%. Although rare, complete or partial PMR is a serious complication which can lead to rapid clinical deterioration and death.^1,2^ A great deal of foresight is essential for an early recognition of this condition, especially in uncommon scenarios, like the case reported. Moreover, the patient was submitted to thrombolytic therapy and prolonged air travel in the acute phase of MI, which could have contributed to additional ischemia and injury.

Transthoracic echocardiography (TTE) is of critical importance in the evaluation of patients in cardiogenic shock after MI, and so it is the ini­tial imaging modality used. It has a sensitivity of 65-85% for the diagnosis of PMR.^3^ However, in some cases TTE is insufficient to accurately ascertain the mechanism causing mitral regurgitation, so an additional characterization with transoesophageal echocardiogram (TEE) becomes crucial to establish diagnosis. TEE can offer superior visibility and characterization of the posterior structures - such as mitral valve apparatus - with a diagnostic yield between 95% and 100%.^4,5^ Although partial PMR may be more difficult to identify than complete rupture, it should always be closely investigated in the setting of a flail/prolapse mitral leaflet.^5^ In our case, only TEE evaluation provided a full characterisation of mitral valve structure and an accurate identification of the mitral valve apparatus disarray. Due to the adverse hemodynamic complications associated with PMR, emergent identification and treatment are essential to improve patient outcomes. The natural history of post-MI PMR is extremely unfavourable under medical treatment alone.^6^ Partial PMR is also considered a surgical emergency as most of the cases will progress to complete rupture.^7^ In our case, the instability of the sub-valvular apparatus was notorious with potential imminent complete rupture, as can be seen in Figure 1.

Bioprosthetic valves are advantageous over mechanical ones due to their comparatively lower incidence of thromboembolic events and avoidance of long-term anticoagulation. Clinically significant bioprosthetic valve thrombosis (BPVT) is considered a rare phenomenon, however accumulated evidence suggests that it is an under-recognised complication.^8^ Its diagnosis remains challenging due to a general lack of awareness on this condition. A combination of clinical and echocardiographic features is helpful for diagnosis. Specific predisposing factors to BPVT include low cardiac output, left atrial dilatation, prior history of thromboembolic events, atrial fibrillation and hypercoagulability. New-onset acute heart failure symptoms, progressive dyspnoea, new thromboembolic event and regression of heart failure symptoms with anticoagulation therapy should be considered as flags for this condition. Some echocardiographic features support the diagnosis of BPVT, such as: direct visualisation of valve thrombosis, like the reported case; a 50% mean gradient increase compared with post-operative evaluation; increased cusp thickness (>2 mm), especially on the downstream aspect of the BPV; abnormal leaflet mobility; regression of BPV abnormalities with anticoagulation, usually within 1-3 months of its initiation or reduced leaflet motion in a cardiac CT scan.^8,9^ The optimal treatment of BPVT remains a matter of debate. The strategy depends on clinical presentation, patient's hemodynamic status, presence of BPV obstruction and valve location. Conventional treatment options include surgery, fibrinolysis and anticoagulation, but anticoagulation coupled with surgery remains the mainstay of treatment.^10^ Although independence from long term anticoagulation is an advantage of bioprosthetic valve replacement, cases like the one we described highlight the importance of considering this condition even in patients without significant risk factors, who display heart failure symptoms early after valve replacement. Post-operatively, patients must be categorised according to risk, and perhaps long-term anticoagulation should be considered for high risk patients, as well as periodic echocardiographic evaluation of biological prosthetic valves. In both complications described in this case, echocardiographic characterization with 2D/3D images was essential for the establishment of a correct diagnosis and for guiding treatment.

This case illustrates two uncommon cardiac mechanical complications, being peculiar their association in the same patient. Despite their distinct pathophysiology, both conditions represent cardiac emergencies requiring a high index of suspicion and an accurate diagnosis. Cardiovascular imaging stands as an extremely valuable supporting technique in a critical-care setting. The precise recognition of the partial papillary muscle rupture (occasionally a missed diagnosis) and the early obstructive bioprosthetic valve thrombosis allowed a prompt and successfully surgical correction of these conditions, with significant impact on patient's health and recovery.
